# α-Linolenic Acid Suppresses Proliferation and Invasion in Osteosarcoma Cells via Inhibiting Fatty Acid Synthase

**DOI:** 10.3390/molecules27092741

**Published:** 2022-04-24

**Authors:** Huijin Fan, Wenyuan Huang, Yong Guo, Xiaofeng Ma, Jianhong Yang

**Affiliations:** 1 Savaid Medical School, University of Chinese Academy of Sciences, Beijing 101400, China; fanhuijin09@mails.ucas.ac.cn; 2 National Research Institute for Family Planning, Beijing 100081, China; 3 College of Life Sciences, University of Chinese Academy of Sciences, Beijing 100049, China; huangwenyuan16@mails.ucas.ac.cn

**Keywords:** fatty acid synthase, α-linolenic acid, osteosarcoma, apoptosis, metastasis, endoplasmic reticulum stress

## Abstract

Fatty acid synthase (FASN) is highly expressed in multiple types of human cancers and is recognized as one of the targets for treating cancer metastasis. α-Linolenic acid is an omega-3 essential fatty acid and it possesses various biological activities. The present study was designed to reveal the effects of α-linolenic acid on osteosarcoma and to reveal whether the mechanism of α-linolenic acid in anticancer activity may be related to FASN inhibition. The cytotoxicity of α-linolenic acid was assessed in osteosarcoma MG63, 143B, and U2OS cells. Cell viability was detected by the MTT assay. The protein expression level was detected by western blotting. Flow cytometry, Annexin V/propidium iodide dual staining, and Hoechst 33258 staining were performed to assess the apoptotic effects. Wound healing assay was applied to detect the inhibitory effect of α-linolenic acid on osteosarcoma cells migration. The results showed that α-linolenic acid downregulated FASN expression. α-Linolenic acid inhibited osteosarcoma cell proliferation and migration in a dose-dependent manner. In addition, α-linolenic acid regulated endoplasmic reticulum transmembrane receptors and signal protein expression in osteosarcoma cells. The findings of the present study suggested that α-linolenic acid suppresses osteosarcoma cell proliferation and metastasis by inhibiting FASN expression, which provides a basis as a potential target for osteosarcoma treatment.

## 1. Introduction

Osteosarcoma, which develops from mesenchymal cells, is the most common type of bone malignant tumor, and has a high fatality rate. Most patients with osteosarcoma are children, adolescents, and individuals who are >55 years old [1]. Among all patients with osteosarcoma, adolescents aged 10–20 years account for 60% of cases, and the mortality rate in children and adolescents accounts for 8.9% of cancer-related deaths [2]. It has been reported that patients receiving multi-drug therapy increases the 5-year survival rate to 55–80% [3,4]. However, despite the improvement in prolonging survival rates, patient prognosis remains poor. The five-year survival rate of patients with metastasis is <20% [5,6]. Controlling metastasis has great significance for the treatment of osteosarcoma. Therefore, finding effective ways to inhibit osteosarcoma proliferation and metastasis may be useful for developing a therapeutic approach in the clinic.

Fatty acid synthase (FASN) is a key enzyme involved in the de novo synthesis of long-chain fatty acids, which is speculated to be essential for mammalian endogenous lipid synthesis. As previously reported, fatty acid metabolic pathways are associated with carcinogenesis [7]. FASN is highly expressed in various types of human tumors. However, FASN is expressed at low levels in normal human tissues, except for liver and adipose tissues [8]. Similarly, the expression of FASN in human osteoblasts is much lower than that in osteosarcoma cells [4] According to some studies, inhibiting FASN in vivo or in vitro may suppress the proliferation of cancer cells [9,10]. Various studies have shown that FASN inhibitors such as epigallocatechin gallate (EGCG), C75, and cerulenin could specifically induce cancer cell apoptosis [11,12]. Therefore, FASN is regarded as an antitumor target, which is related to the survival of cancer cells. However, whether FASN could be a therapeutic target for osteosarcoma remains unknown.

The endoplasmic reticulum (ER) is the major site involved in lipid metabolism by various enzymes [13]. Homeostasis through the ER accumulation of misfolded or unfolded proteins in the ER lumen is destroyed. Continuous changes in the extracellular environment and ER surroundings cause ER stress. In response to ER stress, a series of signaling pathways of the unfolded protein response (UPR) are activated [14,15].

Through signaling pathways, there are three ER transmembrane sensors that mediate the UPR: (i) Inositol-requiring enzyme 1 (IRE1); (ii) protein kinase R-like endoplasmic reticulum kinase (PERK); and (iii) activating transcription factor 6 (ATF6) [16]. The activation of UPR pathways leads to autophagy to restore ER homeostasis. If homeostasis is not restored, continuous ER stress can induce cell apoptosis and programmed cell death to remove damaged cells [17]. However, the role of FASN inhibition-induced ER stress, which is associated with osteosarcoma cell apoptosis, still remains unclear.

α-Linolenic acid is an 18-carbon polyunsaturated fatty acid with a double bond at the nine, 12, and 15 sites. It is an essential polyunsaturated fatty acid for the human body. Since the human body cannot synthesize it, it can only be obtained from food. Studies have found that α-linolenic acid has a variety of functions, which can reduce the incidence of stroke and coronary heart disease by inhibiting the formation of atherosclerosis and also effectively improving neural cognitive ability [18,19]. α-Linolenic acid also has a good effect on obesity and non-alcoholic fatty liver disease. Studies have found that adding α-linolenic acid to the diet of mice can significantly reduce the obesity rate in mice, and human experiments have also shown that there was a decrease in body fat and body weight in subjects with α-linolenic acid intervention. α-Linolenic acid has a good effect in the treatment of tumors [20,21]. Serological analysis shows that the content of linolenic acid in cancer patients is much lower than that of normal people. Increasing the level of linolenic acid in cancer patients has a certain effect on the recovery and treatment of cancer. In addition, studies have also found that α-linolenic acid can activate the AKT-MAPK pathway, thereby inhibiting the growth of tumor cells [22,23].

Thus, the present study aimed to determine whether α-linolenic acid could induce osteosarcoma cell apoptosis and investigate the underlying molecular mechanisms, and to reveal whether the mechanism of α-linolenic acid in anti-cancer activity may be related to FASN inhibition. These results might provide new novel clues for the treatment of osteosarcoma treatment.

## 2. Results

### 2.1. α-Linolenic Acid Inhibits the β-Ketoacyl Synthase (KS) Domain of FASN

**Figure 1:**
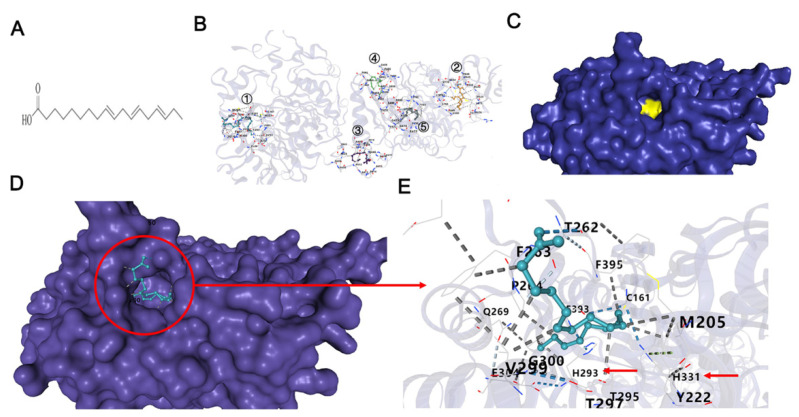
Molecular docking by means of computer simulation also showed that α-linolenic acid acted on the β-ketoacyl synthase domain of FASN. (**A**) Chemical structure of α-linolenic acid. (**B**) Five binding models of α-linolenic acid with KS. ① Vina score of −6.1, ② Vina score of −5.9, ③ Vina score of −5.7, ④ Vina score of −5.3, ⑤ Vina score of −4.9. (**C**) KS domain and the catalytic center are shown in the yellow area. (**D**) α-Linolenic acid binding the hydrophobic pocket on the KS domain. (**E**) Details of KS binding with α-linolenic acid.

Molecular docking by means of computer simulation proved that α-linolenic acid acted on KS of FASN. The top five binding models are shown in Figure 1B, where the best Vina score was −6.1, which represents the high affinity of α-linolenic acid with KS domain in position ①. Interestingly, the catalytic center consisting of C161 H293 H331 was located at this position in the yellow area in Figure 1C. α-Linolenic acid could block the hydrophobic pocket by interacting with the surrounding amino acids, which formed a broad hydrophobic interaction. Coincidentally, Ac–CoA needs to pass this hydrophobic pocket pathway to enter the catalytic center to be catalyzed. In the presence of α-linolenic acid, the catalytic triad was blocked and Ac–CoA was inhibited to enter the catalyzed center, which resulted in decreasing the FASN activity (Figure 1).

### 2.2. α-Linolenic Acid Reduces the Viability of Osteosarcoma MG63, 143B and U2OS Cells

**Figure 2:**
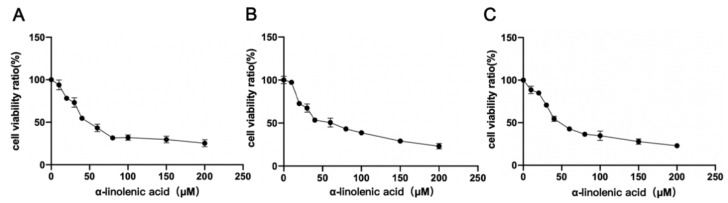
Effect of α-linolenic acid on the viability of osteosarcoma MG63, 143B, and U2OS cells. Cell viability was then determined using an MTT assay. (**A**) MG63, (**B**) 143B, and (**C**) U2OS cells were treated with 0, 10, 20, 30, 40, 60, 80, 100, 150, and 200 μM α-linolenic acid for 24 h. The ratio of α-linolenic acid-treated cells to control cells was taken as the percentage of cell viability. The data are presented as the mean ± SD of three independent experiments. FASN, fatty acid synthase.

An MTT assay was used to identify whether α-linolenic acid inhibited the viability of osteosarcoma MG63, 143B, and U2OS cells. Compared with the control (0 μM), α-linolenic acid reduced cell viability. α-Linolenic acid showed a high degree of inhibition of cell proliferation in a dose-dependent manner, and the IC_50_ value was 51.69 ± 0.14 μM in MG63 cells (Figure 2A), 56.93 ± 0.23 μM in 143B cells (Figure 2B), and 49.8 ± 0.50 μM in U2OS cells (Figure 2C).

### 2.3. α-Linolenic Acid Induces Apoptosis of MG63 Cells

**Figure 3:**
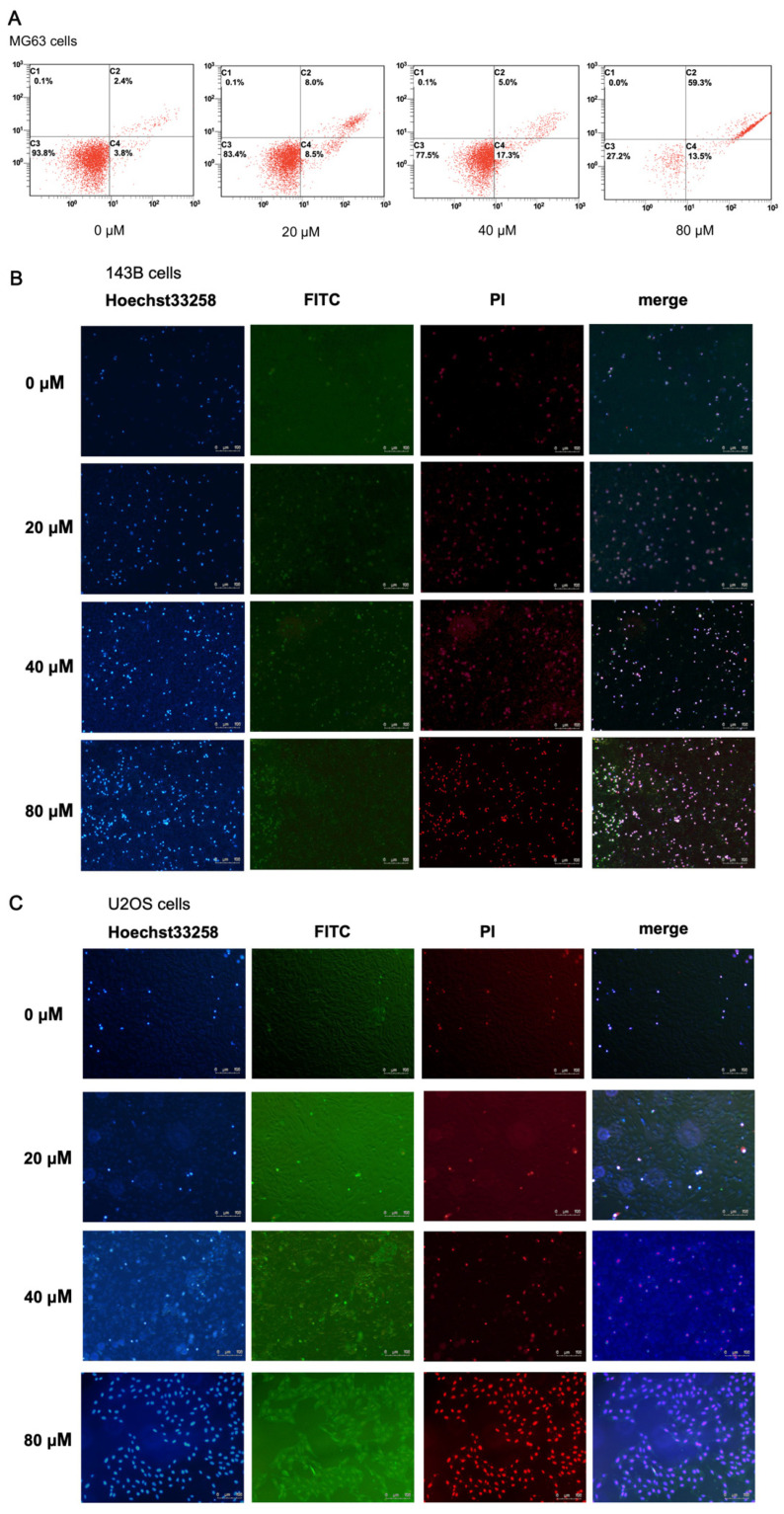
Effect of α-linolenic acid on the apoptosis of MG63 osteosarcoma cells, and 143B and U2OS cells. (**A**) MG63 cell apoptosis was analyzed by flow cytometry with Annexin V and PI double staining. The four regions represent the state of cells: Alive, necrosis, early apoptosis, and late apoptosis. (**B**,**C**) Hoechst 33258 staining and Annexin V/PI double staining were performed in 143B and U2OS cells treated with α-linolenic acid, respectively. The concentrations of α-linolenic acid were 0, 20, 40, and 80 μM.

According to the IC_50_ value, α-linolenic acid (0, 20, 40, and 80 μM) was used to treat MG63, 143B, and U2OS cells to test its apoptotic effect. Following treatment of MG63 cells with α-linolenic acid for 24 h, cells were double-stained with Annexin V and PI and analyzed by flow cytometry. α-Linolenic acid induced the apoptosis of MG63 cells in a dose dependent manner (Figure 3A).

### 2.4. α-Linolenic Acid Induces Apoptosis of 143B and U2OS Cells

The effect of α-linolenic acid on the apoptosis of 143B and U2OS cells was detected by Hoechst 33258 staining and Annexin V/PI double staining. After treatment with α-linolenic acid (0, 20, 40, and 80 μM) for 24 h, the results of Hoechst 33258 staining showed that cell membrane permeability and nuclear concentration increased after treatment (Figure 3B,C). The results of Annexin V/PI double staining were positive for Annexin V-FITC and PI, indicating that cells were in terminal apoptosis or had already died (Figure 3B,C). α-Linolenic acid induced 143B and U2OS cell apoptosis in a dose-dependent manner.

### 2.5. α-Linolenic Acid Reduces the Migration of Osteosarcoma Cells In Vitro

**Figure 4:**
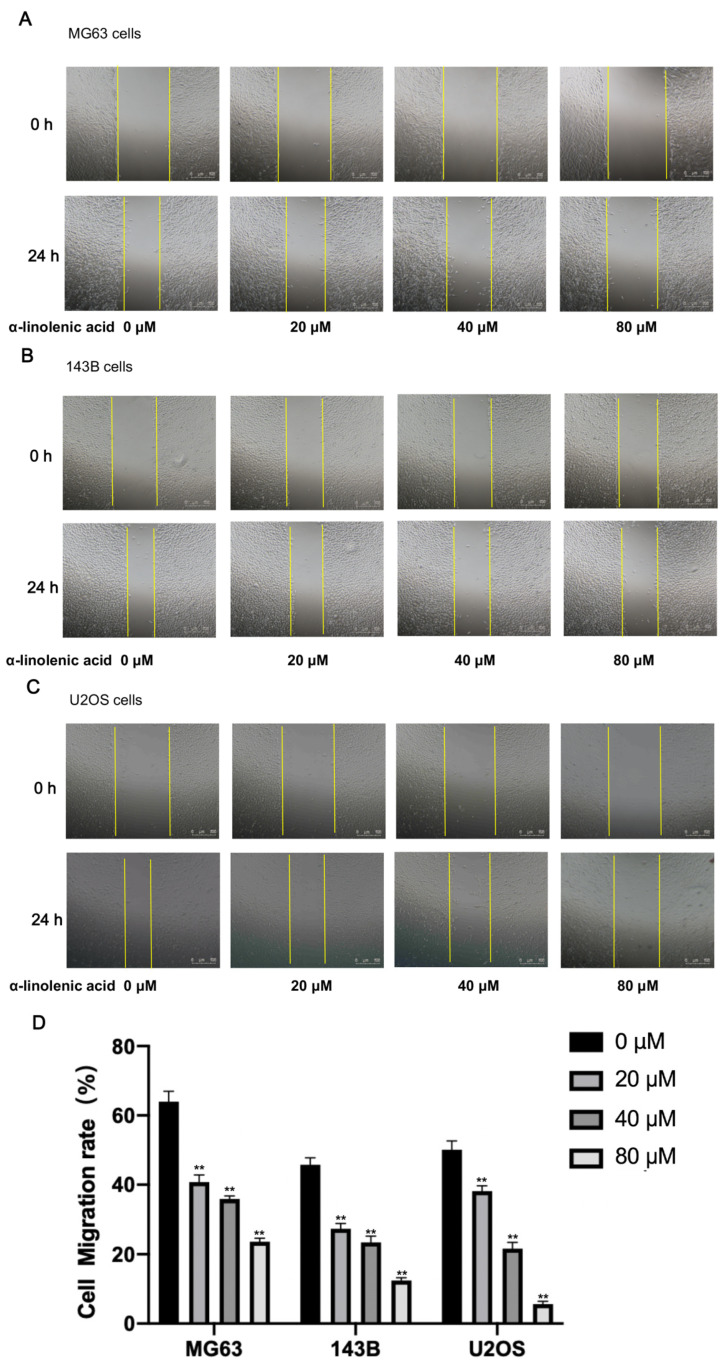
α-Linolenic acid suppresses the migration of MG63, 143B, and U2OS cells. (**A**–**C**) Wound healing assays showed that MG63, 143B, and U2OS cells treated with α-linolenic acid (0, 20, 40, and 80 μM) reduced migration. (**D**) Representative images of migration are shown, and ImageJ was used to analyze the average area of cell migration of MG63, 143B, and U2OS cells in each group. The data are presented as the mean ± SD of three independent experiments. ** *p* < 0.01.

The effect of α-linolenic acid on osteosarcoma cell migration in vitro was measured by performing wound healing assays. Compared with the untreated control group, wound healing was decreased after α-linolenic acid treatment (Figure 4). These findings provide evidence that α-linolenic acid participates in inhibiting the migration of osteosarcoma cells in a dose-dependent manner.

### 2.6. α-Linolenic Acid Downregulates FASN Expression in Osteosarcoma MG63, 143B Cells

**Figure 5:**
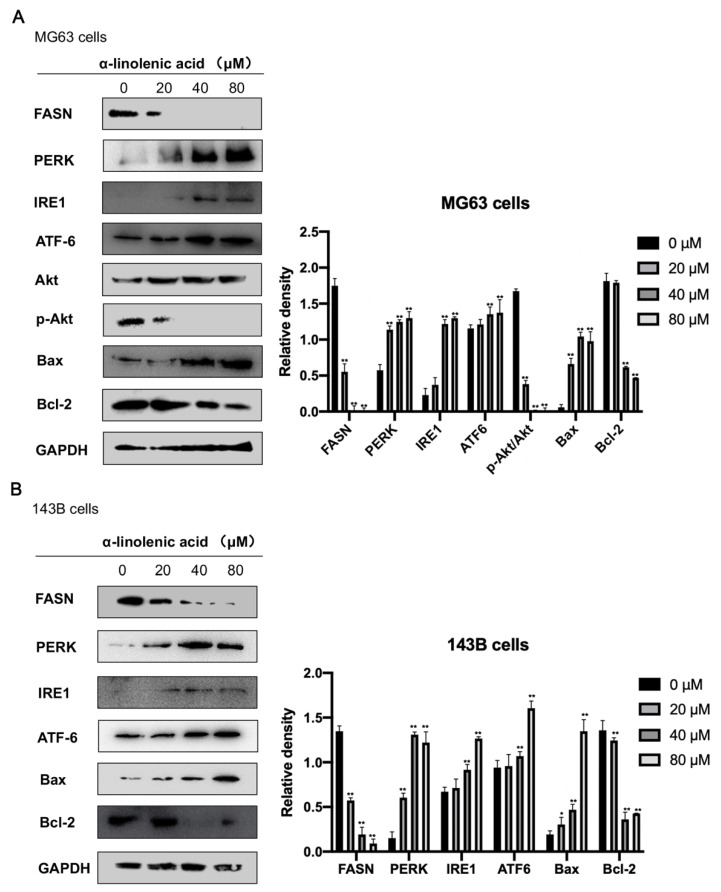
α-Linolenic acid regulates the protein expression levels in osteosarcoma MG63 and 143B cells. (**A**) MG63 cells were treated with α-linolenic acid for 24 h, and then the expression levels of FASN, PERK, IRE1, ATF6, p-Akt, Akt, Bcl-2, and Bax were analyzed by western blotting. (**B**) 143B cells were treated with α-linolenic acid for 24 h, and then the expression levels of FASN, PERK, IRE1, ATF6, Bcl-2, and Bax were analyzed by western blotting. Using GAPDH as a reference band, these results were semi-quantified using ImageJ. The concentrations of α-linolenic acid were 0, 20, 40, and 80 μM. The data are presented as the mean ± SD of three independent experiments. * *p* < 0.05, ** *p* < 0.01. FASN, fatty acid synthase; p-, phosphorylated; ER, endoplasmic reticulum; PERK, protein kinase R-like endoplasmic reticulum kinase; IRE1, inositol-requiring enzyme 1; ATF6, activating transcription factor 6.

α-Linolenic acid is a highly active FASN inhibitor. However, no previous studies have focused on the effect of α-linolenic acid on FASN activity in osteosarcoma cells. MG63 and 143B cells treated with α-linolenic acid (0, 20, 40, and 80 μM) reduced the expression of FASN in a dose-dependent manner compared with the control (Figure 5).

### 2.7. α-Linolenic Acid Induces ER Stress in Osteosarcoma MG63 and 143B Cells

The expression levels of UPR markers (IRE1, PERK, and ATF6) were detected by western blotting to determine whether α-linolenic acid induced ER stress in osteosarcoma MG63 and 143B cells. As shown in Figure 5, cells were treated with α-linolenic acid (0, 20, 40, and 80 μM) for 24 h, which led to significantly increased expression levels of IRE1, ATF6, and PERK. The results showed that α-linolenic acid could cause ER stress in human osteosarcoma cells.

### 2.8. α-Linolenic Acid Downregulates Akt Phosphorylation in Osteosarcoma MG63 Cells

The effect of α-linolenic acid on the activity of Akt was determined. After MG63 cells were treated with α-linolenic acid for 24 h, the cells were collected, lysed, and analyzed by western blotting to detect phospho-Akt. α-Linolenic acid reduced the phosphorylation of Akt (Ser 473) in a dose-dependent manner, but did not affect total Akt expression. The western blotting results indicated that the p-Akt/total Akt expression ratios were decreased significantly in α-linolenic acid-treated MG63 cells compared with the negative control (Figure 5A).

### 2.9. Effects of α-Linolenic Acid on Bax and Bcl-2 Protein Expression in Osteosarcoma MG63, 143B Cells

Bax is a pro-apoptotic protein. Increased levels of Bax can promote cell apoptosis and play a key role in cell apoptosis induced by mitochondrial stress. Bcl-2 is an anti-apoptotic protein that can inhibit the release of cytochrome c-induced apoptosis. Decreased Bcl-2 protein levels may lead to cell apoptosis. After α-linolenic acid treatment, the expression levels of Bax increased in a dose-dependent manner. In contrast, the expression levels of Bcl-2 decreased in a dose-dependent manner (Figure 5).

## 3. Discussion

According to reports, the expression levels of FASN in cancer cells is considerably higher than that in normal cells [10,11]. Studies have shown that FASN is a dual target for the treatment of obesity and cancer. FASN is a key enzyme for the synthesis of long-chain fatty acids in vivo. In recent years, studies have shown that fatty acids biosynthesized by FASN are used to satisfy cell division and proliferation. FASN expression is associated with the prognosis of patients with malignant tumors. FASN plays a key role in the occurrence and spread of numerous types of malignant tumors and is an attractive target for cancer treatment [24,25]. FASN inhibition has become a promising target for cancer treatment, and various inhibitors (EGCG, Curcumin et al.) have been studied [26,27]. Osteosarcoma is the most common primary malignant tumor in children and adolescents. At the time of diagnosis, 30% of patients with localized osteosarcoma and 80% of patients with metastatic osteosarcoma will exhibit recurrence of the cancer. Lung metastasis is one of the main causes of death in patients with osteosarcoma [28,29,30,31]. In the present study, it was hypothesized that FASN could be a potential target of osteosarcoma. To the best of our knowledge, the present study is the first to show that α-linolenic acid can inhibit the proliferation and metastasis of osteosarcoma via inhibition of FASN. This may highlight novel ideas for the treatment of osteosarcoma.

Studies have shown that dietary α-linolenic acid intake is associated with a lower risk of death from all causes, cardiovascular disease, and coronary heart disease, and a slightly higher risk of death from cancers [21]. α-Linolenic acid can induce cell apoptosis in human breast cancer cells [22,23]. Therefore, α-linolenic acid is speculated to prevent metastasis and subsistence of cancer cells. KS domain is an important part of FASN, which could catalyze acetyl-CoA with malonyl-CoA to produce 3-ketoacyl-CoA. Consequently, FASN activity could be decreased directly by inhibiting KS domain. As the interaction of the KS domain with α-linolenic acid, the FASN ability of synthesizing fatty acid could be suppressed in the present of α-linolenic acid. However, Liver X Receptors (LXR) and Peroxisome Proliferator-activated Receptor α (PPARα) could be regulated by fatty acids, furthermore LXR and PPARα could regulate FASN expression directly [32,33]. Accordingly, α-linolenic acid inhibits FASN activity by binding with the KS domain, which leads to the expression of LXR and PPARα being inhibited, and then FASN expression was decreased by α-linolenic acid. In the present study, by using computer molecular docking technology, it was found that α-linolenic acid could bind to the KS region of FASN (Figure 1). In addition to α-linolenic acid inhibiting the activity of FASN, this research proved that it could also inhibit the expression of FASN (Figure 5). The study revealed that α-linolenic acid regulated cell growth through FASN (Figure 2). Therefore, it is reasonable that the decrease in FASN expression precedes the inhibition of cell growth, which is also supported by our results. Furthermore, α-linolenic acid treatment induced a dose-dependent reduction in the viability of MG63, 143B, and U2OS cells. Additionally, this study proved that α-linolenic acid inhibited the growth and metastasis of osteosarcoma cells. It was demonstrated that α-linolenic acid suppressed the migration of osteosarcoma cells through the wound healing migration assay (Figure 4). Additionally, this study proved that α-linolenic acid inhibited the growth and metastasis of osteosarcoma cells (Figure 4).

One way to treat cancer is inducing the apoptosis of cancer cells. In the current study, the relationship between α-linolenic acid-mediated apoptosis and the inhibition of FASN was explored. Flow cytometry, Hoechst 33258 staining, and Annexin V/propidium iodide (PI) dual staining are all commonly used methods to detect cell apoptosis. The results of flow cytometry, Hoechst 33258 staining, and Annexin V/PI double staining showed that α-linolenic acid induced apoptosis in a dose-dependent manner (Figure 3). The pro-apoptotic and anti-apoptotic activities of Bcl-2 family proteins are controlled by cell death signals on the mitochondria [34,35]. In the present study, it was found that the administration of α-linolenic acid reduced the expression of anti-apoptotic Bcl-2 and increased the pro-apoptotic Bax protein in MG63 and 143B cells (Figure 5). These results indicated that the apoptosis induced by α-linolenic acid was associated with the Bcl-2 family, indicating that α-linolenic acid induced apoptosis in osteosarcoma cells.

ER stress is closely related to lipid metabolism. α-Linolenic acid affects the synthesis of fatty acids by inhibiting FASN expression, which induces ER stress [13,14]. Cells can induce self-protection signal transduction pathways to deal with ER stress, known as the UPR (PERK, IRE1, and ATF6) [15,16]. However, if the ER stress is severe and lasts for a long period of time, the UPR will eventually initiate the cell apoptosis pathway. In the current study, α-linolenic acid induced cell apoptosis through the phosphatidylinositol 3-kinase (PI3K)/Akt pathway, and the Bcl-2 family proteins played a critical role. These results showed that α-linolenic acid mediated ER stress and cell apoptosis.

It was speculated that the molecular mechanisms associated with the involvement of FASN in osteosarcoma proliferation and metastasis may be related to ER stress activation and the PI3K/Akt pathway. One of the most common changes in human cancer cells is Akt activation [36]. Inhibition of FASN activity can decrease cell proliferation and induce apoptosis in cancer cells [37]. Some reports have shown that PI3K/Akt signaling plays an important role in the development of cancer [38]. According to previous reports, FASN inhibition can reduce the proliferation and migration of colorectal cancer cells by suppressing the activity of the “HER2-PI3K/Akt axis” [39]. Conversely, Akt inhibition demonstrates a similar downregulation effect on FASN mRNA and protein expressions in vitro [40]. In the present study, when treated with α-linolenic acid, p-Akt expression was significantly reduced in MG63 cells. It was also found that α-linolenic acid decreased osteosarcoma cell proliferation and migration. These results indicated that α-linolenic acid regulated FASN expression to affect the activity of the PI3K/Akt axis.

**Figure 6:**
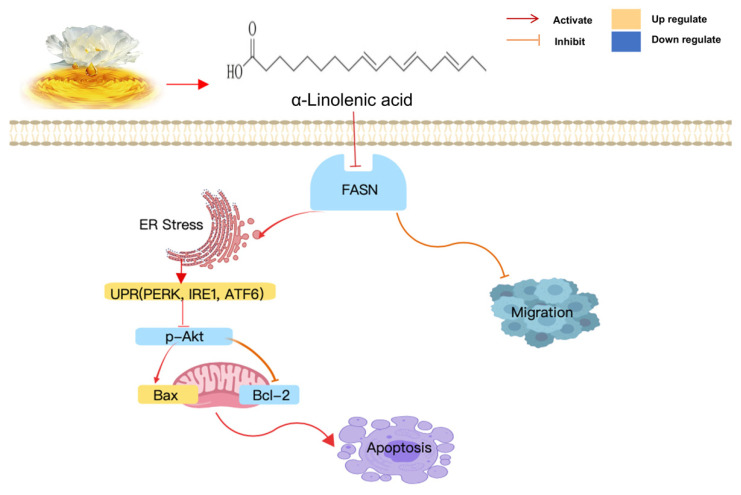
Working hypothesis of the anticancer effects of α-linolenic acid in osteosarcoma cells.

In the present study, it was revealed that α-linolenic acid could bind to the KS region of FASN and downregulate FASN expression. It was also demonstrated that α-linolenic acid suppressed the proliferation and migration of osteosarcoma cells. α-Linolenic acid could induce ER stress, which is related to cell apoptosis. These phenomena indicated that osteosarcoma cell proliferation and metastasis may be associated with the inhibitory effect of α-linolenic acid on FASN expression (Figure 6). Thus, FASN may be a potential target for the treatment of osteosarcoma, and α-linolenic acid could be an adjuvant drug for osteosarcoma therapy. We hypothesize that the signaling pathways will affect each other. Thus, the molecular mechanisms will be further studied and in vivo experiments will be performed to reveal the anti-metastatic properties of α-linolenic acid in tumor growth and metastasis, and determine whether FASN is a promising target and prognostic predictor for treating osteosarcoma.

## 4. Materials and Methods

### 4.1. Reagents

DMSO, α-linolenic acid, and BSA were purchased from Sigma-Aldrich (Merck KGaA, Darmstadt, Germany). Dulbecco’s modified Eagle’s medium (DMEM) and fetal bovine serum (FBS) were purchased from Biological Industries (Kibbutz Beit-Haemek, Israel). Antibodies against FASN (C2065), GAPDH (#5174), Phosoho-Akt (Ser473#4060), and Akt (#4691) were purchased from Cell Signaling Technology, Inc. (Beverly, MA, USA). Antibodies against Bax (ab32503), Bcl-2 (ab32124), IRE1 (ab124945), PERK (ab229912), and ATF6 (ab227830) were purchased from Abcam (Boston, MA, USA).

### 4.2. Molecular Docking Simulation of α-Linolenic Acid with FASN

Docking is particularly useful in studying drug target discovery. In this project, CB-DOCK [41] was used to explore the binding affinity between α-linolenic acid and KS, one of the FASN domains. KS structure (3HHD) and α-linolenic acid structure were downloaded from the PDB database and PubChem database, respectively.

### 4.3. Cell Lines and Culture

Human osteosarcoma cell lines MG63, 143B, and U2OS are commonly used in osteosarcoma research and have some differences in their proliferation rate. These three cell lines were used in this study, which were purchased from The Cell Bank of Type Culture Collection of The Chinese Academy of Sciences (Shanghai, China) and were STR authenticated. The cells were grown in DMEM supplemented with 10% FBS in a humidified atmosphere containing 5% CO_2_ and maintained at 37 °C.

### 4.4. Cell Viability Assay

MG63, 143B, and U2OS cells were seeded at a concentration of 1 × 10^6^ cells/mL and cultured in 96-well plates until they reached confluence, and then the media were replaced with serum-free media containing increasing concentrations of α-linolenic acid (0, 10, 20, 30, 40, 60, 80, 100, 150, and 200 μM) and cells were incubated for 24 h (37 °C, 5% CO_2_). The concentration of solvent was consistent, and the influence of solvent was excluded. Subsequently, the media were replaced with fresh media containing 0.5 mg/mL MTT. Following incubation for 4 h at 37 °C, the plate was poured out again and 150 μL DMSO was added to dissolve the formazan crystals present in living cells. The plate was analyzed by spectroscopic analysis using a microplate spectrophotometer (synergy2; BioTek Instruments, Inc., Santa Clara, CA, USA) at a wavelength of 492 nm. The data were taken from an average of five wells, and the measurement was repeated three times.

### 4.5. Immunoblot Analysis

Cells were washed with cold PBS three times, and RIPA lysis buffer containing 1 mM PMSF was added to lyse the cells on ice for 5 min. The lysate was centrifuged in an Eppendorf tube at 13,000× *g* for 30 min at 4 °C, and the supernatant was taken as the cell lysate. After measuring the protein concentration using the BCA method, SDS-PAGE was used to isolate the same amount of total protein extract (20 μg), then following electrophoresis, proteins were transferred to PVDF membranes. The membrane was then incubated with the primary (FASN, Phosopho-Akt, Akt, Bax, Bcl-2, IRE1, PERK, ATF6, and GAPDH) and secondary antibodies for ≥1.5 h in Tris-buffered saline containing 5% BSA. After incubation, the membrane was washed with Tris-buffered saline containing 0.1% Tween-20. ECL was used to detect the signals, and GAPDH was used as the internal control to compare the target protein levels. These results were semi-quantified with ImageJ software (Version 1.0, National Institutes of Health, Bethesda, MD, USA), and then calculated.

### 4.6. Detection of Cell Apoptotic Rates by Flow Cytometry

According to the manufacturer’s protocols, the Annexin-V-FITC Apoptosis Detection Kit (BD Biosciences, Franklin Lakes, NJ, USA) was used for MG63 cell apoptosis detection. Cells were harvested after drug treatment for 24 h. The cells were washed three times with cold PBS, and then 1 × 10^6^ cells/mL were resuspended in 1× binding buffer. Subsequently, 500 μL cell suspension was incubated with 5 μL Annexin-V-FITC and 10 μL PI in the dark for 15 min, and analyzed using a FACSCalibur flow cytometer (BD Biosciences) over 1 h. An excitation light at a wavelength of 488 nm was used, and green fluorescence (FITC, wavelength 530 ± 30 nm) and red fluorescence (PI, wavelength > 620 nm) were detected. Apoptotic cells were determined by Annexin V+/PI− (early apoptotic cells) and Annexin V+/PI+ (late apoptotic cells).

### 4.7. Hoechst 33258 Staining

143B and the U2OS cells were seeded at a concentration of 1 × 10^6^ cells/mL and cultured until they reached confluence in 24-well plates. Cells were treated with specified doses of α-linolenic acid (0, 20, 40, and 80 μM). After incubation at 37 °C in a 5% CO_2_ incubator for 24 h, the culture medium was replaced with medium containing 0.5 μM Hoechst 33258. The cells were incubated for 20 min, and then washed with PBS three times. A fluorescence microscope was used to examine nuclear staining, and images were assessed using ImagePro Plus software (Version 6.0, Media Cybernetics, Inc., Pittsfield, MA, USA).

### 4.8. Annexin V/PI Dual Staining Microscopy

143B and U2OS cells were seeded at a concentration of 1 × 10^6^ cells/mL and cultured until they reached confluence, followed by treatment with the indicated doses of α-linolenic acid (0, 20, 40 and 80 μM). Cells were stained with fluorescein-coupled Annexin V and PI stain for 5 min in the dark. The apoptotic cells were measured under a fluorescence microscope, and images were analyzed using ImagePro Plus software (Version 6.0, Media Cybernetics, Inc., Pittsfield, MA, USA). The results were calculated using ImageJ.

### 4.9. Wound Healing Assay

Cells were seeded at a concentration of 1 × 10^6^ cells/mL, cultured in 6-well plates, and incubated to 70–80% confluence. Then, a 10 μL plastic pipette was used to make vertical scratches to form a ‘wound’. The culture medium was removed and replaced with fresh DMEM without FBS. Cells were treated with different concentrations of α-linolenic acid (0, 20, 40, and 80 μM) and incubated at 37 °C for 24 h. Images were captured under an inverted microscope. ImageJ was used to measure the wound area. The area of fold-change and the cell migration rate were calculated. Each area was counted in three fields and repeated three times. These results were quantified with ImageJ, and then calculated.

### 4.10. Statistical Analysis

Statistical analysis among three or more groups were determined by one-way ANOVA with Tukey’s post-test using Origin 8.5 software. All data are presented as the mean ± SD of three independent repeats. *p* < 0.05 was considered to indicate a statistically significant difference.

## 5. Conclusions

α-Linolenic acid, via downregulating FASN expression, induced human osteosarcoma MG63, 143B, and U2OS cell apoptosis in a dose-dependent manner. FASN inhibition may have a notable effect on altering the expression of key proteins, which may influence tumor progression and migration. These results support the idea that α-linolenic acid, as a FASN inhibitor, plays a role in regulating ER transmembrane receptors PERK, IRE1, and ATF6. α-Linolenic acid also regulated signal protein expression. These findings indicate that FASN may serve as a therapeutic target in osteosarcoma and that α-linolenic acid may be a health care product for osteosarcoma therapy.

## Data Availability

The data presented in this study are available on request from the corresponding author.
